# Nurses’ challenges when supporting the family of patients with ALS in specialized palliative home care: A qualitative study

**DOI:** 10.1080/17482631.2023.2238984

**Published:** 2023-07-25

**Authors:** Alexandra Beyermann, Margareta Asp, Tove Godskesen, Mirkka Söderman

**Affiliations:** a Division of Caring Science, School of Health, Care and Social Welfare, Mälardalen University, Eskilstuna, Sweden; b Palliative Research Centre, Department of Health Care Sciences, Marie Cederschiöld University, Stockholm, Sweden; c Centre for Research Ethics & Bioethics, Department of Public Health and Caring Sciences, Uppsala University, Uppsala, Sweden; d Senior Lecturer, Division of Caring Science, School of Health, Care and Social Welfare, Mälardalen University, Eskilstuna, Sweden, ORCID, 0000-0001-5391-623X

**Keywords:** ALS, caregivers, family, home care nursing, nurses experience, palliative care, relatives, qualitative

## Abstract

**Purpose:**

Being a family member to someone who has amyotrophic lateral sclerosis (ALS) is demanding and often requires sacrificing a lot. Family members can experience fatigue, anxiety, guilt and need support. The aim was to explore registered nurses’ (RNs’) experiences of providing support to the families of patients with ALS within specialized palliative home care (SPHC).

**Methods:**

A qualitative explorative design. Interviews were conducted with RNs (*n* = 11) from five SPHCs in Sweden and analysed using qualitative content analysis.

**Results:**

The results emerged in the following categories:”To support in an increasingly difficult everyday life”, based on the sub-categories: “Creating a trusting relationship”, “Balancing between the needs of patients and their families”, and “Sharing knowledge about dying to the families”;”To support in emotionally challenging situations”, based on the sub-categories: “Harbouring family members’ difficult feelings”, “Providing support even though the situation is unpleasant” and “Being able to give support by receiving confirmation and support from others”.

**Conclusions:**

RNs working in SPHC have an important role in providing support in several ways to the families of patients with ALS, through facilitating their everyday life and giving emotional support when needed, based on the needs of both patients and the families.

## Introduction

Amyotrophic lateral sclerosis (ALS) is a progressive, incurable, neurodegenerative disease, and leads to death usually three to five years after diagnosis (Norris et al., 2020). It is estimated that almost half a million persons worldwide live with ALS, and most are between 40 and 70 years old at the time of diagnosis with an average age of 55 years. ALS is also 20% more common in men than in women. In Sweden, about 250 persons are diagnosed with ALS each year, and 850 live with the disease (National Board of Health and Well-fare, 2018). Patients with ALS often suffer from severe bodily symptoms such as pain and weakness, muscle cramps and degenerations, speaking, swallowing and breathing difficulties, as well as anxiety, fear, and affected cognitive ability (Norris et al., 2020). In a late stage of the disease, the person with ALS may become completely dependent on others. Persons with ALS express specific and significant losses across personal, social, and professional relationships, as well as physical and functional losses and various future consequences of loss such as not being part of their families’ lives in the future (Soundy & Condon, 2015). Also, living with ALS decreases the sufferers’ autonomy, and creates fear (A. Ozanne & Graneheim, 2018; A. O. Ozanne et al., 2013). Patients’ fear can include fear of severe symptoms as well as having to receive breathing assistance (Oh & Schepp, 2013). The patients may also see the diagnosis as unfair, causing an existential crisis (Soundy & Condon, 2015). Furthermore, becoming a burden to one’s family and facing their anxiety can be experienced as guilt-filled and tough (A. O. Ozanne et al., 2013). In this paper, family includes spouses or other family members who share their daily life with the person with ALS.

Family is an important prerequisite for the patient to be cared for and eventually die at home (Danielsen et al., 2018). The family members may, at the beginning of the disease, have a lot of energy to support their loved ones. Often, this energy decreases as the disease progresses, with increased need for support. It is common for family members of ALS patients to refrain from working, leisure, and social interaction to support in practical matters and to ensure the safety of their loved ones (de Wit et al., 2019). Family members’ social life is also limited by the patient withdrawing from their social life (Anderson et al., 2019), which is further complicated by the fact that patients can prefer to be cared for by the family members (de Wit et al., 2019). They often experience a high burden of care (Burke et al., 2015), which can lead to a low quality of life (Galvin et al., 2020). They can feel traumatized, powerless, and in despair due to the tasks they need to perform. They can find it difficult to find solutions and live a bearable everyday life (Winther et al., 2020). Moreover, they may feel guilt when they long for a break and rest (Oyebode et al., 2013). Family members may fear that the disease may progress quickly. Their fears may also be related to a preconceived notion of the disease that may be obtained through media portrayals (A. Ozanne & Graneheim, 2018). Spouses of persons with ALS have an especially higher mortality in the form of accidents and suicide than family members of patients with other diagnoses (Kläppe et al., 2021). The families situation can be eased by support from healthcare or if they have the idea that it is a matter of time because death is imminent (de Wit et al., 2019).

Persons with advanced ALS are in need of extensive palliative care interventions, usually from home healthcare (Norris et al., 2020). They are usually connected to an expert team at the hospital when they receive their diagnosis. However, due to the adoption of the principle “care at home” in Sweden, persons in need of care are encouraged to live at home with support as it helps them remain independent for as long as possible. For persons with ALS, it may take a long time before they are provided specialized palliative home care (SPHC), and often only when the disease has advanced significantly. Palliative care is active care with a holistic view that includes persons of all ages, with serious suffering related to serious illness, and especially for persons near the end of life (International Association for Hospice and Palliative Care, 2018). In addition to the fact that healthcare aims to promote the quality of life for the persons suffering from a life-threating disease and their families, the intention is to prevent and alleviate the suffering through early detection, careful analysis and treatment of pain and other physical, psychological, social, and existential problems. Palliative care at home is complex, based on conditions that differ between different homes and families (Wu et al., 2022), such as opportunities to adapt the home to care or the ability of family members to care for their loved ones. Palliative care is dependent on the resources being adapted to specific needs, having a multi-professional team, and on creating relationships with both patients and their families. This work is both emotionally demanding and emotionally rewarding for registered nurses’ (RNs), as it includes being both responsible for patients’ care, ensuring patients’ best possible quality of life and supporting family members in their situation and their grief (Pusa et al., 2015). The RNs need to have different skills and competencies, to find strategies that can work in the different situations that arise (Alvariza et al., 2020). The work also includes eliciting the families’ needs and wishes for support, which can be achieved through having good relationships and trust (Pusa et al., 2015). This can be performed through communication and continuity, being available and working in multi-disciplinary teams towards common goals (Josefsson et al., 2018). RNs’ professional roles and experience can create security for both patients and their families.

RNs providing palliative care in the patient’s home, can be related to the theorist Watson’s (Watson, 2008) description of the transpersonal care relationship. This theory offers a framework for this study as it delves deep into the interpersonal level, describing the core of a nurse’s caring work and providing insight into how nurses care. Caring, according to Watson, promotes a holistic approach to the individual, emphasizing integrity and where the core of caring consists of respect for each other’s human dignity. Further, human caring begins when the nurse enters another person’s lifeworld. In the relationship, the nurse tries to protect, strengthen, and maintain the patient by helping “the other” (e.g., a patient or relative) find respite despite illness, suffering, and pain as well as increasing the other’s self-awareness, control, and their own healing ability.

As ALS is a relatively rare disease, only a few persons with ALS are cared for in palliative home care units yearly. Consequently, there is limited knowledge regarding palliative care and support specifically tailored for persons with ALS and their families in home care setting. The care of patients with ALS requires detailed planning and major efforts, while also requiring sensitivity to the needs of both the patients and their families (Lerum et al., 2017). Consequently, to be able to improve RNs’ knowledge and skills and the life situation for both the families and, by extension, patients with ALS, more research is needed. Therefore, the aim of this study was to explore RNs’ experiences of providing support to the families of ALS patients within SPHC.

## Materials and methods

The study has a qualitative and exploratory design to describe the phenomenon of the individual experiences that were studied, and where new knowledge in this specific area was sought. In March 2017, RNs working within SPHC were invited to this study regarding their experiences of supporting the families of patients with ALS. Interviews with included RNs were conducted during May to June 2017. This study follows the COnsolidated criteria for REporting Qualitative research (COREQ) checklist.

### Setting and participants

In Sweden, SPHC is offered to patients with complex symptoms or life situations and in need of advanced palliative care. The RNs included in the present study, worked in multi-professional teams, henceforth referred to as SPHC teams with specific knowledge and expertise in palliative care, consisting of physicians, RNs, physiotherapist, occupational therapist, and hospital social workers. RNs are the professional category that besides nursing also act as coordinators for care in the team and have the most contact with patients and their families. In addition to the SPHC team, these patients and the families often have social care and support in the form of personal assistants and home care. Although the care units were largely designed similarly, there was a difference in team size and geographical distance to the patients. For example, in larger cities teams were larger and had more patients, while in sparsely populated areas, teams were smaller with fewer patients.

The RNs included in this study, had limited experience in caring for and providing support to patients with ALS and their families. This due to the relative rarity of ALS diagnosis and the fact that patients with ALS are not always cared for at home. Furthermore, within the SPHC, RNs were responsible for the care both for mechanical ventilated and non-ventilated patients in the later stages of the disease.

We used a purposive sample to select the RNs who could provide informative descriptions of the phenomenon in question and thereby serving as a basis to address the research aim. The home care units were randomly selected, and inclusion criteria for participation in the study were RNs working within SPHC who had experience in caring for patients with ALS and providing support to their families. An information letter with a consent form and requests for participation were sent to operational managers responsible for SPHC teams in central and northern parts of Sweden, and in total, five SPHC teams accepted participation in the study. Four of the teams provided care at home around the clock, and one of the teams worked office hours Monday to Friday but collaborated with district nurses with around the clock service. The operational managers who approved the study, provided the information letter and the consent form to RNs who worked in the respective teams.

### Data collection

After receiving the consent forms from RNs interested in participation, the first author arranged the time and places for the individual interviews. An interview guide with seven semi-structured questions (Table I) was designed by the first and third author, based on previous research and interview questions used in similar studies. First, two pilot interviews were conducted, and since the questions were modified to create more openness without affecting the content of the questions, these interviews were included in the analysis. The first author conducted the interviews, according to the interview guide and follow-up questions to gain a deeper understanding of the RNs’ experiences. RNs were asked about experiences, thoughts, feelings, and perspectives related to supporting the families of patients with ALS and were encouraged to describe concrete situations and examples. The interviews took place in a separate room at the RNs’ workplaces. Before interviews began, RNs were informed that participation was voluntary, and that they could cancel their participation at any time without giving a reason. RNs were also informed that the data would be treated confidentially. After interviewing the RNs who consented to the study, data saturation was achieved. The interviews varied between 21–50 minutes (mean 32 minutes) and were recorded on tape.

**Table I.** The interview guide with semi-structured questions used in the study.

1. Can you please tell me about your experiences of supporting relatives of ALS patients?
2. How do you identify the needs of the relatives?
3. What kind of challenges have you encountered when supporting relatives?
4. What do you think is particularly important when you encounter relatives in their home?
5. Do you think there is any difference in what relatives of ALS patients need support in compared to other patient groups?
6. Can you tell about a situation in which you felt was important when you were giving support to relatives?
7. Is there something that I haven’t asked you about but that you want to tell me?

### Analysis

A manifest qualitative content analysis according to Graneheim and Lundman (Graneheim et al., 2017) was chosen to describe the RNs’ experiences of supporting the families of patients with ALS. The interviews were transcribed and read several times to gain a deeper understanding of the content. Meaning units, consisting of sentences or longer paragraphs, referring to the aim of the study, were extracted from each individual transcript of interview. These meaning units were condensed and abstracted into a code without changing the meaning of the text. The codes with common content were divided into content areas. The different content areas were compared to each other to create subcategories, and categories points out the manifest content of the transcripts. To ensure that codes were divided into the right context, they were checked against the meaning units and the raw data. The analysis stopped at a manifest level to portray what RNs wanted to convey but with a high degree of abstraction (Graneheim et al., 2017). An example of the analysis process is presented in Table II. Validation of the analytical framework took place through discussions between the authors, and credibility was achieved through discussions between the authors throughout the analysis process via research group meetings and with a larger group of researchers at the university. After analyses, the RNs were given the results in the form of an oral presentation.

**Table II.** An example of the analysis process.

Meaning unit, a sentence or a paragraph	Condensed meaning unit	Code, abstracted and shortened meaning unit	Subcategory, codes from common content areas	Category, the manifest content of the transcripts
“ … that it is very good to talk to our hospital social worker… but she is a support, I think, for us RNs because she can guide both how to ask questions, how to think, why I feel the way I do about it and… it makes my attitude towards the patient easier that I get some tools, I feel.”	Needs and receives support and confirmation through conversations with colleagues in the team	Receives support and confirmation from colleagues	Being able to give support by receiving support	To support in emotionally challenging situations

### Ethical considerations

Approval for the study was obtained through the ethical board at Marie Cederschiöld University. The participants were informed that participation was voluntary, and that the information collected would be handled in such a way as to ensure that only authorized persons had access to it. Further ethical aspects were considered in line with the Declaration of the World Medical Association in Helsinki (World Medical Association, 2014) and The Swedish Privacy Protection Authority GDPR (The Swedish Privacy Protection Authority DPA, 2016).

## Results

In total, 11 RNs consented to participate in the study, 10 women and one man (Table III), and they were aged 41 to 61 years (median 50 years). They had worked as RNs 7 to 29 years (median 21 years), had various specialist training, and had 4 to 21 years of experience of working at SPHC, including experience in both caring for patients with ALS and providing support to their families (median 7 years).

**Table III.** Characteristics of the registered nurses.

	Median (min-max)
Age	50 (41–61)
	**n**
**Sex**	
Women	10
Men	1
**Specialist training**	
Specialist nurse in palliative care	1
District nurse	2
Diploma in palliative care	1
Courses in palliative care	22
**Years in the profession**	
5–10	2
11–15	3
20–25	5
<25	1
**Years worked in palliative care, including experience of care and support for patients with ALS and the family**	
1–5	4
6–10	2
11–15	3
16–20	1
<20	1

(*n* = 11) working at specialized palliative home care teams.

The categories and subcategories revealed after the qualitative analysis that describes RNs’ experiences of supporting the families of ALS patients, are presented in Table IV.

**Table IV.** An overview of categories and subcategories found in the qualitative analysis, that describes registered nurses’ (RNs’) experiences of supporting the families of ALS patients.

Category	Subcategory
To support in an increasingly difficult everyday life	Creating a trusting relationship
	Balancing between the needs of patients and their families
	Sharing knowledge about dying with the families
To support in emotionally challenging situations	Harbouring family members’ difficult feelings
	Providing support even though the situation is unpleasant
	Being able to give support by receiving confirmation and support

### Category: To support in an increasingly difficult everyday life

The RNs provided support in the everyday life of the families of patient with ALS in various ways. They protected their integrity by not intruding into their sphere, balanced the support by listening and seeing the needs and acting when appropriate. The RNs created an arena where the patients with ALS and their families could meet and discuss as spouses instead of patient and carer, and despite different perceptions of the situation, they were living in. It was important for the RNs to convey knowledge and information both to the families of patients with ALS and home care staff to facilitate the families’ situation.

#### Subcategory: Creating a trusting relationship

RNs experienced that patients with ALS were admitted to the SPHC at a late stage of the disease when they already had lost several bodily functions and were therefore dependent on the families. But many of the family members had already cared for their loved one without support for a long time. RNs described that they often observed that the family had developed strategies to deal with everyday life which required a lot of energy and made them very tired, which they ignored. The RNs experienced that even though they perceived that the families of patients with ALS needed relief, they could not reach them and suggest relief measures. They also experienced that if they initiated the relationship by proposing measures that the families did not consider necessary, they would violate the families’ integrity. As one RN described:

He is completely attached to her, but he wouldn’t see it that way and I wouldn’t dare to say it either, because it would have become a violation of course. (Participant 8)

The RNs experienced it as important to establish a relationship based on trust with the families of patient with ALS before approaching their sphere. The relationship was established in several ways, such as giving the patient good care, being responsive to the needs of the entire family, working based on the wishes of the entire family, and showing the family that they saw them and what was happening around them. As one RN stated:

I think the support for them (the family) can be anything from… that I think above all, to show that you see what you see. (Participant 1)

The RNs described that they needed to get to know the patient with ALS and their families to provide support in an adequate way. One way to do that was to always pay attention to them, to ask them how they felt or how they perceived their situation. The RNs also experienced that by caring for the patients in a respectful way, then this contributed to having a good relationship with the family. During most home visits, the RNs were able to find time to talk with the families, either in front of the patients or in a separate room. Sometimes the conversations could be about something else than the patient and the disease, conversations about life in general over a cup of coffee, as one nurse described:

… so we try to talk a bit socially and about all other issues too… so it’s not just that… we… take tests or give medicines so we can talk about much wider things with family members with… depends on… sometimes … they are worried and how will it be with finances, what do I do next, with (other) family members or with the house or how do I solve it, so you try to talk a little as a person with a person, not just as a RN. (Participant 2)

The RNs experienced that encountering the families of patient with ALS as human to human was important in being able to create a relationship based on trust.

#### Subcategory: Balancing between the needs of patients and the families

The RNs experienced that to facilitate everyday life for the families of patient with ALS, they had to deal with the patients and their families expressing different needs and several circumstances going on at the same time. The RNs described that the families confided that their loved ones required so much attention that they themselves rarely had time of their own, time to leave their home, eat meals in peace and quiet or sleep properly. Based on the information the RNs received, they experienced it as important to suggest relief to the families, so that the families could have time for their own needs, both for basic human needs, as well as to meet friends and do activities outside the home. As one RN said:

Because they are tired, because they are exhausted when we have come, when we have come in. So that you need, really…make sure they eat, make sure they sleep, that they go out and get fresh air. (Participant 3)

The RNs experienced that despite these conversations when the families of patients with ALS could describe their situation in words, they could still find it difficult to accept suggested help. It was a balancing act to inform the family that they must get help to cope, offering adequate relief, and at the same time confirm the families’ care efforts as good and important for the patients. The RNs described that they listened and respected the families’ wishes, and the support they could give in such situations was to stand by, wait and keep a watchful eye on the patients. The same RN continues:

The challenge is to maybe see that they can keep going until they collapse…sometimes…the family members. But…they have been shown that we are here when they collapse…that we are here…You go on your journey, but we are here. (Participant 3)

The RNs experienced that sometimes when the families of patients with ALS wanted to get relief, they were prevented by their loved ones. The RNs experienced that the patients were often strong individuals who could control the whole home without realizing their own limitations. One RN described that the patient could sometimes be selfish and did not see how tired the family member was and completely convinced that outside help was not necessary:

The one who gets sick, I think, finds it difficult that…they become quite selfish….the ones I’ve had anyway…that they think about themselves…they don’t think so much about the family…around them and then it will also be a difficult balancing act where how far we can draw the limits, when we see that the family members are almost collapsing because they can’t take it and then there is a sick person sitting next to them and saying no we don’t need help yet, we can handle this … a while longer … . (Informant 10)

The RNs experienced that in such difficult situations, they had to balance and relate to both the patients’ and the families’ experience. The RNs described it as important to give the families both emotional support and empathy, while they also needed to respect the patients’ wishes. By handling different circumstances in parallel, the RNs meant that they became a bridge builder between the patients and the families, which they experienced as an important part of the process of supporting them. Thus, they were able to be open to difficult conversations by highlighting and pointing to what was troublesome and awkward. In this way, the families and the patients with ALS together could put words to their thoughts and difficult feelings, and the RNs, as a third person in the room, could de-dramatize the tension.

#### Subcategory: Sharing knowledge about dying to the families

The RNs experienced that as the disease progressed, the patients with ALS’ and their families’ lives changed more and more, and their homes turned into a mini hospital with lots of assisting tools, where several strangers worked. The home no longer was a home where recovery and rest were natural. Everyday life at home was not the same as before when home care staff or personal assistants who cared for the patients were constantly there. One RN described it as:

Their everyday life is not their own anymore. (Participant 4)

The RNs experienced it as important to respect the home and behave as if they were guests. The RNs described that they observed everyday life becoming increasingly difficult for the families and tried to make it easier by providing information to them. In addition, by informing the home care staff or the personal assistants about the illness and the care that the patients needed. Also, about the importance of being observant of the families’ needs as the RNs described it as important that, for example, spouses could maintain their private lives, to have time together without being disturbed. As one RN described:

So that’s what we try to convey to the assistants, that this is your job. When you notice that the wife comes in and needs to talk, then you ask if it’s okay that we go out for a little while now, that they don’t have to ask for it all the time, but that you can read the situation. (Participant 4)

The RNs described that they had conversations with the families of patients with ALS talking about the disease and the various symptoms that belonged to the disease picture. They described it as important to inform at an early stage that the symptoms that might occur, could be adequately alleviated with medication. Also, to convey that they did not know how the course of the disease should be, but to inform the families about their own experience and competence in this area, including that dying itself was usually peaceful. The RNs experienced that the families were afraid that the patient should die a dramatic death, since they had often received an incorrect image via the media, the internet, or books about what it was like to die from ALS. This concern led the family members to lie awake at nights, listening to the patients’ breathing, which in turn led to even more fatigue. The RNs experienced it as important to discuss this with the family:

There’s also information, that you inform the family about, I think about the latest time yesterday, the last patient, the family members thought what will happen to the patient is that he will suffocate, but that you… the challenge is also that you bring information all the time and preferably as clearly as possible and how often death is usually associated with those who have ALS. (Participant 7)

Through these conversations, which could take place regularly both with the patients with ALS and their families together or individually, the RNs experienced that they could convey security. They experienced it as important to reduce the families’ fear and lift a burden from their shoulders.

### Category: To support in emotionally challenging situations

The RNs cared for the families by showing interest in their situation and by inviting them to conversations. Sometimes the conversations could be of a more tough nature where the families’ feelings of suffering or anger could touch the RNs in different ways. The RNs always remained on the families’ side and provided support through presence and confirmation.

#### Subcategory: Harbouring the family members’ difficult feelings

The RNs described that they invited the families of patients with ALS to individual conversations, but that most conversations took place spontaneously during the home visits. This could happen by the RNs asking a small question or by drawing attention to the families, giving them time and space during the encounter. The RNs experienced that these conversations often took place in a separate room so the patients could not hear the conversation, or in the garden, on the steps outside the house or at the RN’s car. The family members could choose a place where they could talk in confidence and dare to release their mental pressure. The RNs described that during such conversations, the family members could burst into tears, open up, and say that they were tired and couldn’t take the situation anymore. As one RN expressed the importance of listening to the families in such situations:

To be here and now, in their…when they have let go of their…their defences. (Participant 5)

The RNs described being able to learn about things that burdened the families. Sometimes the conversations could be about their feelings of guilt and the shame of being tired while their loved one was dying. The RNs described showing their support by listening and affirming that the families had the right to feel what they felt and to express their feelings, with the aim of reducing their feelings of guilt. The conversations with the families of patients with ALS could also concern their private sphere, such as a wife confiding that she could not respond to her husband’s desire for intimacy because of his bodily symptoms. The RN described her reaction as follows:

I was quiet…I think it’s important…I feel it then anyway, maybe I would have done it differently today or maybe…but then I felt that my absolute…task was to be quiet and just listen and that was actually something which she …felt very, very bad about…and there are no words that I can say that will take this away and then it was better that I be quiet and listen. (Participant 4)

The RNs experienced that sometimes there were no words to say that could lift the families’ burden, on the other hand, the RNs could show respect for the families’ situation by daring to remain in the difficult situation and just listen.

#### Subcategory: Providing support even though the situation is unpleasant

The RNs experienced that they were greeted by the families of patients with ALS in different ways. When the RNs went on a home visit, they did not know what mood the family members would be in. During the same visit, a family member could show several different moods and the RNs had to deal with the relative being happy and then suddenly crying, showing despair or anger. The RNs described that these feelings could be related to the families’ thoughts that the disease progressed quickly, and that they felt powerless and alone, or that they were angry at the disease or at the patients. The RNs experienced in such situations that the family members let the prevailing feeling play out while the RNs were calm and listened. The RNs’ support function in these situations was not to come up with different solutions, but just to listen. Although the family members could change their mood, one RN described that it was easier to support the families who dared to show their feelings than those who were not open:

For the family members who dare to come forward, there you can still be with them, and you can hear their thoughts and you can talk…someone who doesn’t want to talk at all and says they are fine and so somewhere you see that this is not okay. (Participant 5)

The RNs experienced being exposed to unpleasant situations by the families in connection to home visits. It could be that despite hour-long visits to the same patient, their families accused the RNs of having been there too rarely or for too short a time. The families’ frustration could also be about the RNs being there too much. The RNs experienced also that sometimes the families showed the RNs appreciation only to criticize a short while later. Some RNs described that because of the mood swings of the family members, they could sometimes experience discomfort during the home visit and even fear, as one RN described:

Sometimes I was actually a bit afraid of her and not only me but also my colleagues, because she could swing incredibly quickly from being happy and meeting you and thinking you’re the best competent person you’ve ever met, to bringing you down in three red seconds where you felt like the most incompetent person on earth. (Participant 4)

The RNs experienced that even if they sometimes felt discomfort, they understood that this was not directed at them, and in such situations the RNs dared to stay by the families’ side and listen. The support function in this hardship was to receive the families’ frustration and to show the courage to remain in the difficult situation together. The families could also clearly show that they did not like a specific RN and that it took a long time before they accepted that an RN would be responsible for their loved one: ” … yes, but even though they were a bit unpleasant and a bit prickly with me both when I was there …on the phone, I stood out somewhere …I came back…I was there several times a week… (Participant 10). In that case, the RN offered the family member to change RN, but they refused, and despite this response, the RN stayed to support both the patient with ALS and the family.

#### Subcategory: Being able to give support by receiving support and confirmation from others

The RNs experienced that to be able to stand in different situations and provide support to the families of patients with ALS, they needed to fill up with energy through support and confirmation for themselves, through group supervision or discussions with their colleagues in the team. The RNs highlighted the team as an important part of their work. It was important to collaborate with team members and to discuss both medical issues, and approaches to different situations or situations they experienced as tough. Sometimes the RNs experienced that they were being tested when the family members asked the same question several times or to different RNs. Therefore, the RNs experienced that it was good to have discussed with and listened to the team members before entering certain conversations with the families. In this way, they could provide support to the families in a professional manner. The RNs described that after difficult conversations with the families, they needed to put their experiences into words, as it was difficult to carry everything themselves, and to get guidance on how to react to different situations as one RN described:

The fact that we didn’t really know what it is, was dealt with here, so we got support here from the hospital social worker to be able to respond to her feelings, to be able to respond and just be quiet and be able to receive. (Participant 4)

The RNs described that continuing to provide good care to the patients and support to their families, sometimes meant they had to protect their own sphere. It could be that they were in difficult situations with the patients and their families and experienced that they could not bear the burden anymore but needed to stop the ongoing situation to seek support from a colleague. One RN described it as:

… and then I said … I’ll be back in an hour or a half something like that, I went to the car and cried and felt like I can’t handle it…oh then I’m like this no I’ve calmed down, I am talking to a colleague, I had to call and vent … . that this is not my fault, this is a frustration, this is chaos, but now I calm down and can talk to my colleague, I go back and then picked up this thread again. (Participant 5)

The RNs experienced that being able to put into words what they had been through helped them gather strength to continue supporting the families in their difficult situation. Another way to gain strength was through the confirmation they received when they had follow-up conversations with the families after the patients had died, as one RN reflected:

… so then you still understand that even then I have been an important person even though I am absolutely not a relative. (Participant 11)

The RNs described that even the families who showed anger and were critical of the RNs said that it was only after the care period and after the patients had died, that they realized the work the RN had done. The RNs described that the family conveyed that the RNs saw everything that the families of patient with ALS themselves did not see, which was appreciated afterwards.

## Discussion

In this study, we showed that RNs working at SPHC intended to provide support to the families of ALS patients in such a way that they had the families’ best interests in mind. Although the RNs could be faced with difficult challenges when encountering the families, they intended to create conditions for encounters based on trust and understanding. Furthermore, the RNs intended to protect the families’ integrity and dignity.

Admittedly, there are similarities in the experiences of palliative care across patients with different diagnoses. However, most patients with ALS often experience a more extensive and earlier loss in personal, social and professional relationships, as well as physical and functional capabilities, compared to patients with other life-threatening diseases (Soundy & Condon, 2015). In addition, the fear of severe symptoms and the need for respiratory assistance is particularly tangible for both the patient and the family (Oh & Schepp, 2013). The results from the current study show that the RNs experienced that the families of patients with ALS had a high burden of care to which they had gradually adapted and that they themselves did not always see their need for support, a phenomenon also described by Lerum et. al (Lerum et al., 2017). The RNs in our study also experienced that the family members’ role as, e.g., a spouse, could be negatively affected by the heavy burden of care and when the home turned into a hospital-like environment, but by showing respect for their home, the RNs were able to protect the spouses’ private sphere. This can be related to Alvariza et. Al (Alvariza et al., 2020), and their study showing that RNs working in palliative home care see themselves as guests in patients’ homes and that they adapt their way of working to show respect for their home. Our study also shows that the RNs took the spouses to patients with ALS situation seriously and tried to protect them by making the home care staff aware of the spouses’ need to be spouses in their own home. Such actions can also be related to Nyholm et. al (Nyholm et al., 2018), and their study that the carer maintains the families’ dignity by being there and acting as the other’s advocate and protecting their rights. In this way, the carer is also responsible for the ethical promise to the professional role.

In Oh & Schepp’s (Oh & Schepp, 2013) study, it appeared that women who care for their husbands with ALS find that caring for their loved one made it difficult for intimacy between spouses. This also emerged in our study, that RNs in trust were made aware of such difficulties, and that they experienced that in these situations the most important task was to be quiet and just listen. This is also underlined in previous research (Pusa et al., 2015). The RN’s actions can also be related to Watson (2008), who states that when the nurse is authentically present, she shows respect and reverence for the other’s life world. If the other person’s subjective life world is given space, their basic values are accepted, and the RN can contribute to alleviating suffering and promoting health processes.

The results show that the RNs needed to relate to the patients with ALS and the family members’ different stories about the same problem by not taking anyone’s side but by becoming a bridge builder between the patients and their families. Through their presence, the RNs experienced that they were able to support the patients with ALS and their families to raise problems for discussion and in this way give space for the patients and their families to agree on a certain issue. The RNs’ approach can be explained in accordance with Watson (Watson, 2008) in that the RN is aware of her own living space when she enters the patient’s living space and the living space of the family. Thanks to her own awareness of her feelings, the RN can show respect for the living space and privacy of the patient with ALS and the family. Because the RN was not biased, neither the patient nor the family were left out of the mutual community. Instead, the RN invited them both to an encounter where they had the conditions to meet and agree. That RNs have such an approach is confirmed in Josefsson et. al (Josefsson et al., 2018), and their study where RNs considered it important, when they were between patients’ and their families’ different perceptions of the same problem, to have dialogue with both parties without taking sides, and to show that they were present in the moment.

Furthermore, our study shows that RNs experienced that they dared to remain and provide support to the families of patients with ALS despite being faced with challenging situations where the families questioned them, showed frustration, anger, or aggression. The RNs remained in the sensitive area, listened, and provided support by being present in the situation. In Pusa et al (Pusa et al., 2015), they show that RNs who had similar experiences felt emotional stress but understood the vulnerable situation of the families. That RNs in the present study remained and showed reverence in difficult situations can also be explained, in accordance with Watson (Watson, 2008), that these RNs cared about the families and could put themselves aside. Through RNs’ peace of mind and soul, the RNs could be authentically present in encounters with another human being (the relative) and showed reverence for their lifeworld, even though she herself was questioned. Because the RNs listened in without judging, they could in this way contribute to promoting the families’ health processes. The results further showed that to provide this support in a good way, the RNs experienced that they needed to reflect with colleagues in the team (e.g., with hospital social worker) or in supervision. This can be related to a previous study by Danielsen et al (Danielsen et al., 2018). who found that collaboration, reflection in the team and supervision were important for RNs who worked with family support in home care. Further, it can be related to Watson (Watson, 2008) who describes that nurses need to show care for themselves for them to gain increased awareness and develop both as persons and as carers. When the RNs in the present study described that they sought support from colleagues, then this showed they took care of themselves and had an awareness of their own situation. They needed to fill up with energy, partly to protect their own sphere and partly to be able to care. Watson (Watson, 2008) also points out that when the nurse gives herself space to face her feelings, she can face the feelings of others. By the RNs in the present study putting their experiences and feelings into words in conversations with colleagues or in supervision, they built a more stable foundation for continued care.

Our study shows that during follow-up interviews after the death of the patients with ALS, RNs experienced that they received confirmation for the care given, this confirmation also came from the families who had been critical of the RNs during the care period. This can be related to Watson (Watson, 2008) that the RNs, despite difficult situations, protected the privacy of the families. In the encounters with the families, the RNs chose how to behave, precisely to preserve the integrity of the families by remaining in the difficult situation. This decision meant that the families took this with them into the future and could in turn, at a later stage, give the RNs confirmation.

Further, it emerged in our study that the RNs protected the family members’ integrity by setting aside their own need to offer help. Even though the RNs described that they saw that the families of patients with ALS were exhausted, they could wait to suggest helping measures until the families were ready to accept it. Similar ways of acting are described by Pusa et. al (Pusa et al., 2015). when the RNs in palliative home care show that they respect the families’ experiences of care. The RNs’ intention is to create a good relationship with families to create trust in this way. Trust and not trespassing on the sphere of the families was seen as a prerequisite for being able to tailor both support and care. Such an approach can also be related to Nyholm et. al (Nyholm et al., 2018). who state that when the carer has a clear picture of her own mission and believes in the other person, dignity is created in the care. The carer also shows respect for the other person by acting in accordance with her own conscience but also considering the other persons’ wishes, even if these differ from her own. The RNs’ approach in the current study, can also be related to Watson (Watson, 2008), who states that respect for each other’s human dignity forms the core of care. Nursing with human care begins when the nurse enters another person’s living space and begins to discover their spirit and soul. By being authentically present, the nurse shows respect for the other’s dignity and gives space for the other’s subjective life world. In this way, the other’s basic values are accepted. In the authentic presence, the nurse does not abandon the other so that she is excluded from a mutual communion with others. The RNs in our study described how they showed respect for the family members’ life world and gave them space to do what they themselves believed was right. The RNs respected and allowed the families of patients with ALS to go in a different direction while standing and were prepared to receive them when they needed support.

### Strengths and limitations

The RNs in this study were invited via the operational managers, from a total of five SPHC teams. From a research ethics perspective, that procedure was advantageous as those not interested in joining the study were not identified at all. However, the RNs included in this study worked at five different SPHC teams, were of different ages and had been active in the profession for different lengths of time. This breadth in the selection process increased the study validity. The trustworthiness of the study was strengthened by using an analysis method suitable for analysing the RNs’ experiences in supporting the families of ALS patients within SPHC. The data was analysed solely by the first author, which could be seen as a weakness, however, the findings and the meaning-bearing units were discussed by all authors—which gives the analysis strength. Using an inductive approach could be seen as a weakness as it can be an obstacle to gaining new insights and is a result of the researcher’s pre-understanding (Graneheim et al., 2017). On the other hand, pre-understanding can also open the researcher’s understanding of the phenomena. During the analysis process, the research team had ongoing discussions to be aware of the pre-understanding to strengthen the results. Two of the authors already had experience as RNs within SPHC. However, all authors were involved in all aspects of the study, to limit the risk of the pre-understanding. The rich descriptions of RNs’ experiences that emerged in the analysis, can be seen to increase transferability to the families of other diagnostic groups but also other arenas of care. It can be considered a limitation that the RNs’ experiences in this study were based on a small number of cases due to the relative rarity of ALS diagnosis and the fact that not all patients with ALS receive home care. However, it is important to note that despite the limited number of cases, the descriptions provided by the RNs´ yielded rich insights and allowed the phenomenon in question emerged.

## Conclusion

The results of this study show that RNs working within SPHC have an important role when it comes to providing support to the families of ALS patients. RNs enter the family members’ lives and provide support in such a way that they try to make everyday life easier and create meaning in the difficult existence. RNs may need to provide support in situations that are difficult for them, and their support must consider the families’ dignity and integrity.

## Relevance to clinical practice

This study contributes to creating more knowledge about how RNs in SPHC work to provide support to the families of patients with ALS. As ALS is a rare diagnosis, these patients are not often seen within SPHC, and for the same reason, it can take a long time for RNs to acquire a wider experience in this field. Our results make RNs’ experiences visible and can thus contribute to gaining an increased knowledge and understanding among RNs as to how to support the families. However, RNs conditions are often not optimal, for example due to organizational resources, for providing care and support based on this. In addition, attachment of ALS patients often occurs at a late stage, which makes it difficult to create supportive relationships with the families. Therefore, the results of this study can contribute, even to operation managers within SPHCs, in gaining a greater understanding of RNs’ work in supporting the families of patients with ALS.

## Data Availability

The data that support the findings of this study are not publicly available due to ethical restrictions.

## Geolocation information

The study was conducted in central and northern Sweden, in northern Europe.
